# Swept-source optical coherence tomography imaging of macular retinal and choroidal structures in healthy eyes

**DOI:** 10.1186/s12886-015-0110-3

**Published:** 2015-09-17

**Authors:** Jiawei Wang, Xinbo Gao, Wenbin Huang, Wei Wang, Sida Chen, Shaolin Du, Xingyi Li, Xiulan Zhang

**Affiliations:** Zhongshan Ophthalmic Center, State Key Laboratory of Ophthalmology, Sun Yat-Sen University, 54S.Xianlie Road, Guangzhou, 510060 China

## Abstract

**Background:**

To report the thickness of the retina, retinal ganglion cell (RGC)-related layers, and choroid in healthy subjects using swept source optical coherence tomography (SS-OCT).

**Methods:**

One hundred and forty-six healthy volunteers were consecutively recruited for this prospective observational study. Thickness of retina, RGC-related layers, and choroid in the standard early treatment of diabetic retinopathy study (ETDRS) grid were automatically measured using one SS-OCT (DRI OCT-1, Topcon, Japan). The IOL Master (Carl Zeiss Meditec, Germany) was used to measure axial length (AL).

**Results:**

Thicknesses of the average macular ganglion cell complex (GCC) and ganglion cell-inner plexiform layer (GCIPL) were 105.3 ± 9.7 and 78.5 ± 6.2 um respectively. Neither of them was significantly related with sex, age, or AL. Both showed strong correlations with retinal thickness (r = 0.793, *p* = 0.000; r = 0.813, *p* = 0.000, respectively) and with similar topographic distributions within the retina. The thicknesses of retina and GCC/GCIPL in the inner sectors were significantly higher than in the outer sectors of the EDTRS area, while in the same region of the macula, the choroid exhibited completely different patterns of topographic variation. Men had 7.8 um thicker retina and 34.9 um thicker choroid than women after adjustment for age and AL (all *p* < 0.05). Age and AL could significantly influence the choroidal thickness but not the retina (all *p* < 0.05).

**Conclusion:**

Thickness of GCC/GCIPL in healthy Chinese individuals is not dramatically different across gender, age, and AL groups in terms of ETDRS grid, but sex is critical for retinal and choroidal thickness. Choroidal structure (but not retinal) can be significantly influenced by age and AL.

**Electronic supplementary material:**

The online version of this article (doi:10.1186/s12886-015-0110-3) contains supplementary material, which is available to authorized users.

## Background

Various retinal and choroidal pathologies, including age-related macular degeneration (AMD) and choroidal neovascularization (CNV), are among the most common reasons for severe visual impairment and blindness. For this reason, qualitative and quantitative analyses of retinal and choroidal structures are critical for the diagnosis and treatment of vitreo-retinal and choroidal diseases. Optical coherence tomography (OCT), as an essential tool in ophthalmology, can noninvasively capture detailed *in vivo* high resolution images of retinal and choroidal structures. This high resolution has enabled clinicians to accurately measure the thickness of local RGC-related layers, including ganglion cell complex (GCC) [1], ganglion cell-inner plexiform layer (GCIPL) [2], retina and choroid [3].

Three inner retinal layers, including the nerve fiber, ganglion cell, and inner plexiform layer, are collectively known as the GCC [4, 5]. The latter two layers are known as GCIPL. Macular GCC/GCIPL assessment has high sensitivity and early diagnostic value for detecting many ophthalmic diseases. Recent studies have demonstrated that GCC/ GCIPL thickness exhibit accurate detection of preperimetric glaucomatous damage when compared with the circumpapillary retinal nerve fiber layer [1, 6–9].

The Early Treatment of Diabetic Retinopathy Study (ETDRS) chart is considered to be the gold standard in the evaluation of retinal and choroidal structures in the posterior pole, and is widely used in clinical application of ophthalmology. Many studies have reported the normal distribution of data for retinal and choroidal thickness in the ETDRS area [3, 10–13]. However, there is no related information to date about the thickness of GCC/GCIPL in the ETDRS chart with large age and AL span. The first objective of the present study is to evaluate GCC/GCIPL thickness in the ETDRS grid of healthy Chinese subjects.

Cumulative evidence supports the view that choroidal thickness is clearly influenced by sex, age, and axial length (AL) in healthy subjects [12, 14–16]. Whether or not the sex/age/AL-related changes in GCC/GCIPL coincide with those in the choroid remains unclear. Therefore, the second principal objective here is to assess the influence of sex/age/AL on retina, GCC/GCIPL, and choroid. With the advances in OCT technology, a novel system called swept source OCT (SS-OCT) is able to automatically measure the thickness of retina, GCC/GCIPL, and choroid in one scanning. Investigators have published a large number of either retinal or choroidal thickness, while few studies have reported the corresponding thickness of GCC/GCIPL in the same region. As far as we know, this is the first time that baseline GCC/GCIPL thickness has been measured with SS-OCT, and it is also believed to be the first time to evaluate differences in the topographic variation between retina, GCC/GCIPL, and choroid, and correlation between these parameters with sex, age, and AL in healthy Chinese volunteers.

## Methods

The transversal study included 146 healthy Chinese volunteers, recruited from our hospital staff and the students of Sun Yat-sen University, from January 2014 to June 2014. All participants underwent a complete ophthalmic evaluation in the clinical research center at Zhongshan Ophthalmic Center, Sun Yat-sen University, Guangzhou, China, and gave written informed consent after study approval by the Ethical Review Committee of Zhongshan Ophthalmic Center. The study adhered to the provisions of the Declaration of Helsinki for research involving human subjects Additional file 1.

All the study participants were healthy individuals with no history of ocular disease or visual symptoms; aged at least18 years; intraocular pressure (IOP) <21 mmHg; normal appearance of optic nerve head; normal anterior chamber angles; and a best-corrected visual acuity (BCVA) of 1.0 or better. Exclusion criteria included IOP > 21 mmHg; history of intraocular surgery or ocular trauma in the study eye; high myopia or hyperopia (magnitude exceeding ± 6 diopters of spherical equivalent refraction); retinal or choroidal abnormality detected by SS-OCT; poor image quality due to severe cataract or unstable fixation; or severe systemic diseases such as diabetes mellitus, rheumatism, or malignant tumors.

The comprehensive ophthalmical evaluation included: measurement of visual acuity and BCVA; IOP measurement using Goldmann applanation tonometry; slit lamp examination; fundus examination with a 90D lens; measurement of axial ocular dimension using ocular biometry (IOLMaster, Zeiss, Germany); and autorefractometry examinations. Refraction data were converted to spherical equivalents, counted as spherical diopters plus one half of the cylindrical dioptric power. Mean thicknesses of retina, GCC/GCIPL, and choroid were automatically acquired through one SS-OCT scan (DRI OCT-1, Topcon).

**Fig. 1:**
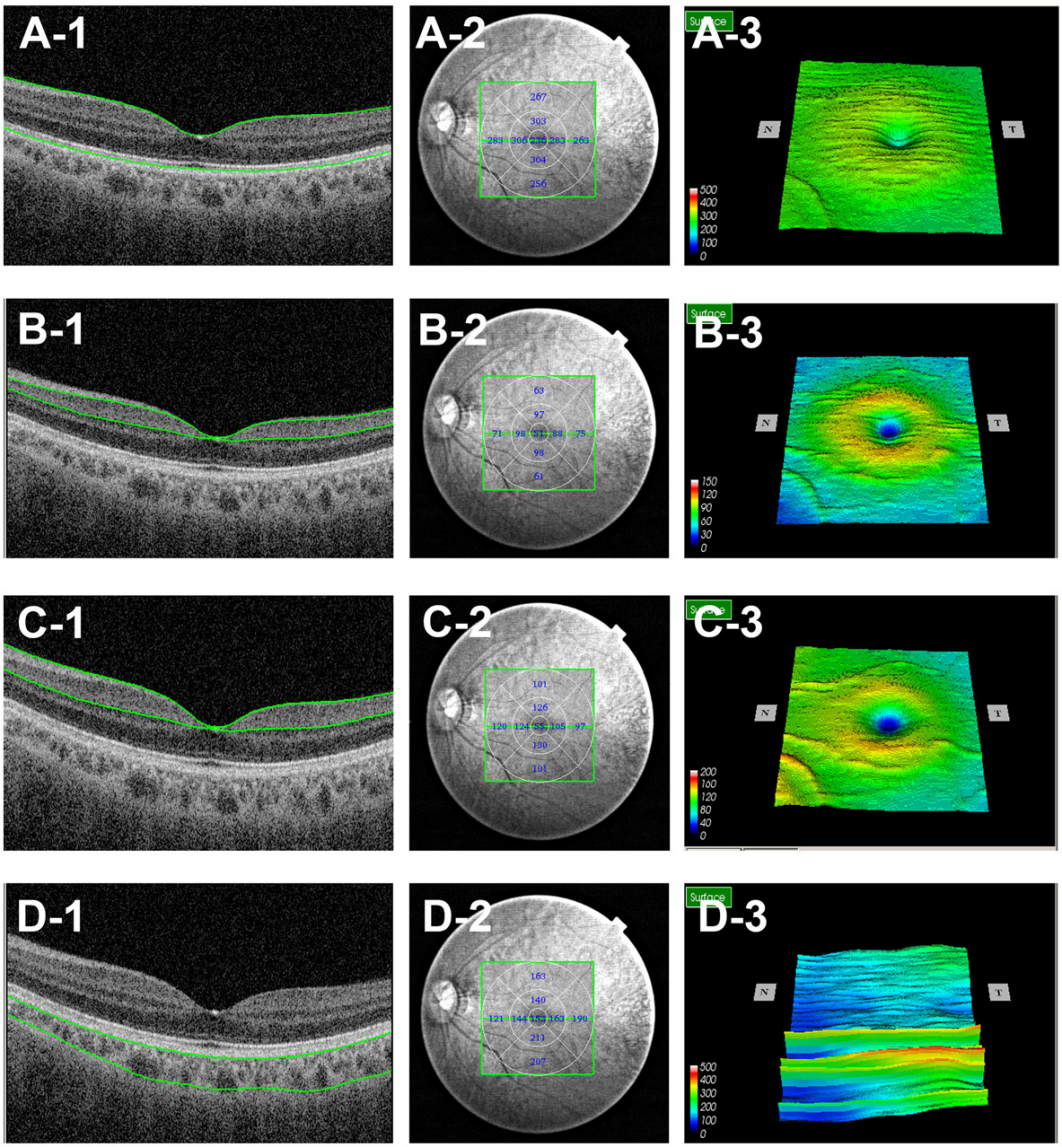
Example of a healthy eye imaged using SS-OCT in the ETDRS area. Retina (A-1), GCC/GCIPL (C-1/B-1), and choroid (D-1) were automatically segmented, and thickness measurements were subsequently calculated through available built-in software (A-2,B-2,C-2,D-2). GCC/GCIPL showed similar topographic distributions with the retina (A-3,B-3,C-3), while in the same region of the macula, the choroid exhibited completely different patterns of topographic variation (D-3). ETDRS = the Early Treatment of Diabetic Retinopathy Study. Delineation of the nine macular sectors: center = within 1000 um of the central fovea; inner ring = 1500–3000 um from the central fovea; outer ring = 3000–6000 um from the central fovea; The inner and outer rings were segmented into four quadrants (inner/outer superior, inner/outer inferior, inner/outer nasal, and inner/outer temporal)

After pupil dilation with 0.5 % tropicamide and 0.5 % phenylephrine, a 3D raster scan protocol with 3 um axial resolution and a speed of 100,000 A-scans per second was performed to acquire the retinal and choroidal thickness map in the macular region (12 × 9 mm) with a central fixation. Next, the thickness maps were overlapped to the EDTRS grid (6 × 6 mm) to obtain the values for each sector [3, 11]. Built-in software was used to automatically calculate thickness values in the modified EDTRS grid [3]. The grid was subdivided into nine independent sectors; the inner and outer rings, with semidiameters of 1500 um and 3000 um, respectively, were segmented into four quadrants(superior, inferior, nasal, and temporal). The central sector was defined as being within 1000 um of the center of the fovea (Fig. 1).

AL was measured by IOL Master, which has been extensively used and is reported to be very precise [17, 18]. Two experienced and well-trained examiners separately performed OCT and axial length measurement at the same time of day (in the morning around 10 AM) in order to exclude diurnal variations during examination of axial length and choroidal thickness [15].

All analyses used SPSS software version 19.0 (IBM-SPSS, Chicago, Illinois, USA). Normally distributed data were expressed as mean ± standard deviation (SD). An independent sample *t*-test was used to compare two different groups where data were normally distributed; a Mann-Whitney *U* test was used when the data showed a non-parametric distribution. Pearson or Spearman correlation coefficients were used for evaluation of bivariate correlations. For comparison of the thickness of different layers at different subgroups according to age and AL, analysis of variance (ANOVA) or a Kruskal-Wallis test was performed. The relationship between macular retinal and choroidal thickness and sex, age and AL was investigated by linear and stepwise multiple regression analysis.

## Results

### Demographic data

The current study included 146 randomly selected eyes from 146 healthy volunteers (100 female and 46male; mean age, 47.9 ± 14.0 years, range, 20–86 years). Seventy-nine left eyes and 67 right eyes were analyzed. The mean AL was 23.5 ± 1.1 mm (range 20.9–26.9 mm).

### Retinal, GCC/GCIPL, and choroidal thickness in healthy subjects

Table 1 shows the actual numbers in each area of the ETDRS map. The mean retinal thickness was 283.3 ± 16.5 um (range 233.8–361.4 um) and demonstrated a distinct topographic variation of the retina. The subfoveal (center) thickness was significantly thinner in all nine independent sectors (*P* < 0.0001). The thickness in the inner sectors was significantly thicker than in the outer sectors. In the inner sectors, the retina was thickest in the inner superior (309.7 um) and was significantly thicker than the other three sectors (*P* < 0.0001). In the outer sectors, the thinnest area was the inferior (267.1 um), while the nasal retina was the thickest (281.6 um), and with significantly differences between the four different regions (*P* < 0.0001).

**Table 1:** Anatomical outcomes of retina, GCC/GCIPL and choroid in the in the standard early treatment of diabetic retinopathy study (ETDRS) charts

	Retina	GCC	GCIPL	choroid
Mean	283.3 ± 16.5	105.3 ± 9.7	78.5 ± 6.2	264.1 ± 105.9
Outer superior	277.2 ± 117.9	111.5 ± 11.3	70.24 ± 6.4	270.1 ± 101.4
Inner superior	309.7 ± 17.3	120.3 ± 11.2	94.75 ± 8.0	263.3 ± 102.2
Outer temporal	275.4 ± 22.7	111.3 ± 18.4	74.5 ± 6.5	244.3 ± 115.8
Inner temporal	304.5 ± 20.4	114.8 ± 11.8	94.4 ± 8.8	267.0 ± 111.9
center	233.4 ± 32.3	45.8 ± 11.1	44.4 ± 14.5	276.7 ± 112.4
Inner nasal	298.2 ± 29.3	105.5 ± 12.8	89.1 ± 12.2	270.1 ± 113.4
Outer nasal	281.6 ± 35.7	110.2 ± 16.7	78.4 ± 8.5	249.0 ± 116.2
Inner inferior	302.5 ± 19.3	117.1 ± 12.7	92.6 ± 8.2	268.5 ± 115.0
Outer inferior	267.1 ± 14.9	109.4 ± 11.5	68.3 ± 6.9	268.0 ± 118.7

**Fig. 2:**
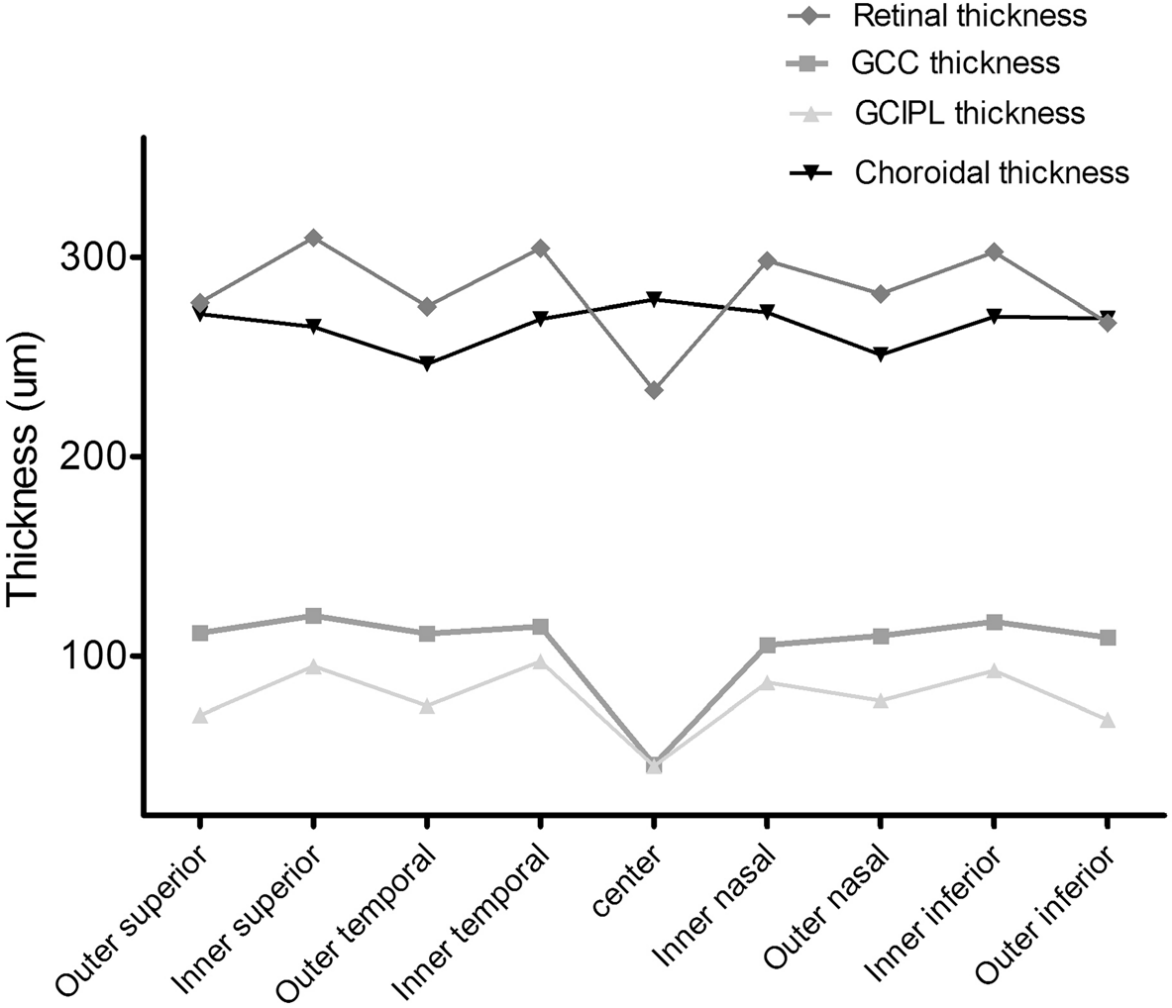
Topographic variation of retinal, GCC/GCIPL and choroidal thickness in the nine ETDRS sectors. GCC/GCIPL showed similar topographic distributions within the retina and the choroid exhibited a completely different distribution pattern

The average macular GCC and GCIPL thickness were 105.3 ± 9.7 um and 78.5 ± 6.2 um in the EDTRS area. Both of these showed strong correlations with retinal thickness (r = 0.793, *p* = 0.000; r = 0.813, *p* = 0.000, respectively) and with similar topographic distributions within the retina in the EDTRS area (Fig. 2). They were found to be perfectly correlated (r = 0.913, *p* < 0.001).

The mean choroidal thickness was 264.1 ± 105.9 um (range 108.9–604 um) in the EDTRS region as automatically measured by SS-OCT. Unlike the retina and GCC/GCIPL, the choroid exhibited a completely different distribution pattern (Fig. 2). Here, the center section (the thinnest sector in the retina and GCC/GCIPL) was the thickest area. Within the inner sectors, the nasal choroidal thickness (270.1 um) was thicker than the inferior (263.3 um), temporal (267.0 um), and superior (263.3 um) quadrants. Within the outer sectors, the thickest area was the superior (270.1 um), followed by the inferior (268.0 um), nasal (249.9 um), and temporal (244.3 um).

### Effects of age, sex, and axial length on the normal retina, GCC/GCIPL, and choroidal thickness

Table 2 shows the influence of sex on the thickness of retina, GCC/ GCIPL, and choroid. Men had a thicker retinal and choroidal thickness than women; mean retinal thickness in men was 7.8 um more than in women, and men had a significantly greater choroidal thickness than women (average 43.2 um thicker = 17.2 % higher in men). However, no statistically significant difference was detected in the thickness of GCC and GCIPL in men as compared to women.

**Table 2:** Mean thickness of retina, GCC/GCIPL and choroid according to gender

	Men	Women	*P*
Retina	289.0 ± 15.9	281.2 ± 15.5	0.006*
GCC	110.4 ± 21.2	105.7 ± 11.0	0.096
GCIPL	79.5 ± 7.5	78.1 ± 5.5	0.271
Choroid	294.1 ± 112.2	250.9 ± 92.4	0.042*

*Independent sample *t*-test

**Fig. 3:**
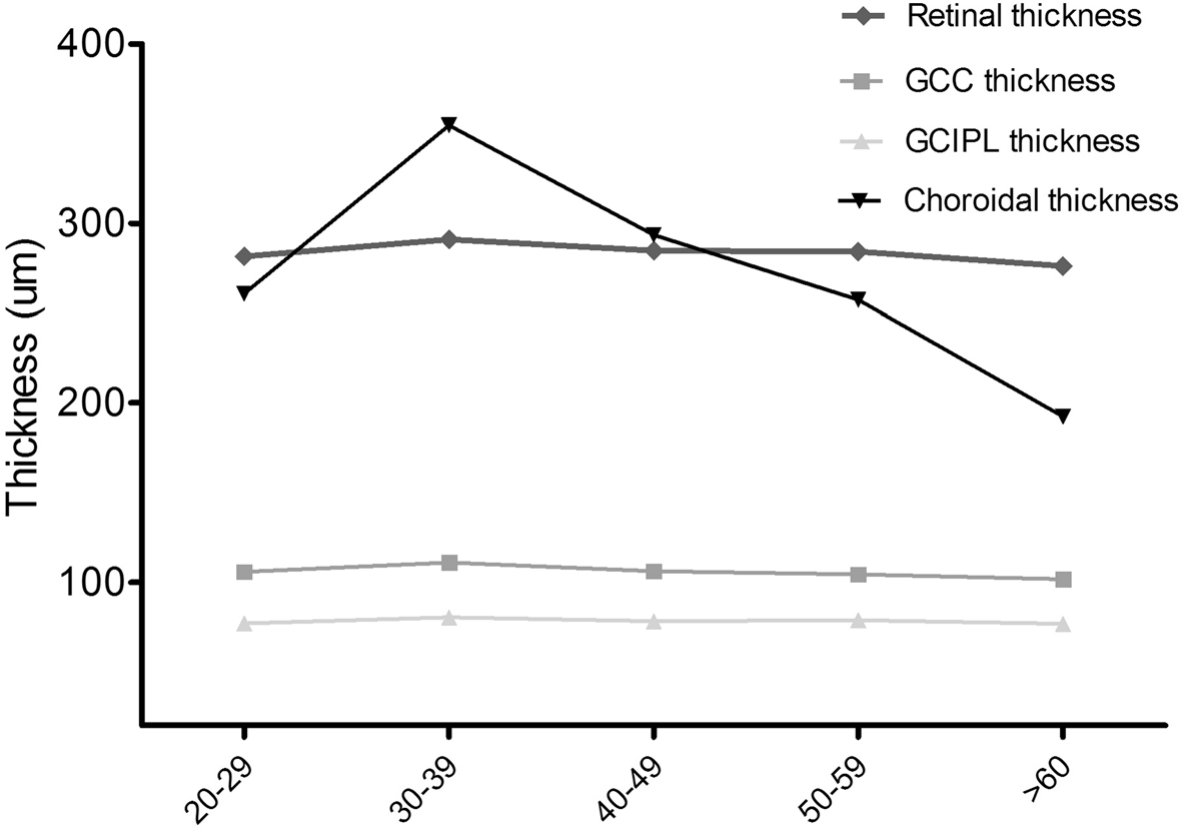
Comparison between retinal, GC/GCIPL, choroidal thickness among age groups. A distinct age-related reduction was found in the choroidal thickness (*P* < 0.001). The choroidal thinning with increasing age seemed to be more rapid when age above 60 years

Table 3 compares the thickness of retina, GCC, GCIPL, and choroid across different ages. Age-related reduction was found in the thickness of retina, GCC, and choroid, especially for choroidal thickness (*P* < 0.001), but it seems that GCIPL thickness does not decrease with age (Fig. 3). Mean thickness of GCIPL in the different subgroups was 77.0 um in those aged 20–29 years, 80.5 um in those aged 30–39 years, 78.3 um in those aged 40–49 years, 78.8 um in those aged 50–59 years, and 76.8 um in those older than 60 years; differences were not statistically significant (*p* = 0.333).

**Table 3:** Comparision of mean thickness of retina, GCC/GCIPL and choroid among different age subgroups

	20-29	30-39	40-49	50-59	>60	F value
n	20	19	27	52	28	-
Reina	281.5 ± 11.5	291.0 ± 17.9	284.7 ± 15.0	284.3 ± 17.4	276.2 ± 16.5	2.591
GCC	105.8 ± 5.9	111.0 ± 13.2	106.3 ± 6.9	104.5 ± 9.8	101.7 ± 10.2	2.916
GCIPL	77.0 ± 4.3	80.5 ± 8.0	78.3 ± 5.1	78.8 ± 7.1	76.8 ± 7.3	1.156
Choroid	260.7 ± 69.3	354.7 ± 141.2	293.5 ± 98.6	257.4 ± 81.5	192.4 ± 95.5	8.914

One-way analysis of variance

**Fig. 4:**
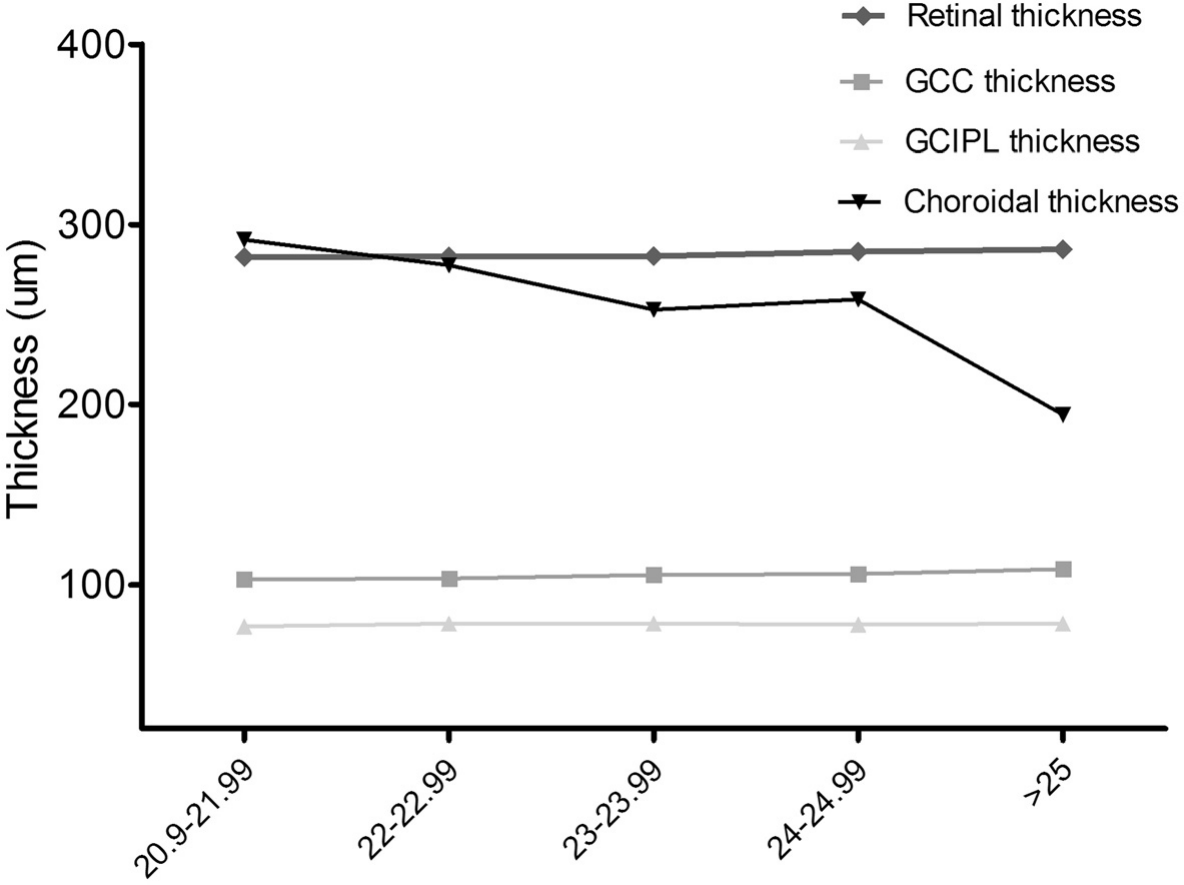
Comparison between retinal, GC/GCIPL, choroidal thickness among AL groups. Choroidal thickness significantly decreased when AL increased to greater than 25 mm. No significant difference was found in the thickness of retina and GCC/GCIPL between different AL subgroups

Table 4 shows that choroidal thickness significantly decreased with increasing AL. When AL increased to greater than 25 mm, mean choroidal thickness decreased to 194.3 um, which was obviously thinner than in emmetropes (AL is assumed to be 22–24 mm, 263.5 ± 99.9 um, *p* < 0.001). There seemed to be no significant difference in retinal thickness, including GCC and GCIPL, between different AL subgroups (Fig. 4). Although there was some variation among different AL groups, this was very small (retina: range from 0.4–4.3 um; GCC: range from 0.6–5.7 um; GCIPL: range from 1.1–1.5 um).

**Table 4:** Comparision of mean thickness of retina, GCC/ GCIPL and choroid among different axial length subgroups

	20.9-21.99	22-22.99	23-23.99	24-24.99	>25	F value
n	6	47	52	20	21	-
Reina	281.9 ± 16.0	282.3 ± 19.6	282.5 ± 15.5	285.0 ± 11.4	286.2 ± 16.8	0.293
GCC	103.0 ± 10.1	103.6 ± 7.6	105.5 ± 9.4	106.1 ± 11.9	108.7 ± 12.2	1.097
GCIPL	76.9 ± 8.0	78.5 ± 6.6	78.5 ± 6.2	78.0 ± 7.5	78.4 ± 7.1	0.093
Choroid	291.5 ± 103.9	277.4 ± 99.5	252.7 ± 99.8	258.5 ± 79.6	194.3 ± 31.1	2.828

One-way analysis of variance

Tables 5 and 6 showed correlations of the measurements with age, sex, and axial length, using linear and multiple regression analysis. No correlation was observed between GCIPL thickness and any of the factors (sex, age, and AL) in either linear or multiple regression analysis. Linear regression analysis showed that AL was negatively correlated with the thickness of GCC.A1mm increase in axial length resulted in a decrease in average GCC thickness of approximately 1.19 um. After adjusting for age and AL with stepwise multiple regression analysis, no significant negative correlation was found between AL and GCC thickness.

**Table 5:** Unadjusted associations between the mean values of retina, GCC/GCIPL and choroid with age and axial length

	Age		Axial length		Sex	
	β(95 % CI)	*p*	β(95 % CI)	*p*	β(95 % CI)	*p*
Retina	-0.153(-0.340,0.034)	0.108	1.333(-1.072,3.738)	0.275	7.891(2.388.13.393)	0.005*
GCC	-0.066(-1.148,-0.017)	0.119	1.193(0.139,2.247)	0.027*	2.704(0.208,5.201)	0.034*
GCIPL	-0.006(-0.079,0.067)	0.876	0.050(-0.888,0.989)	0.916	1.343(-0.843,3.529)	0.227
Choroid	-1.679(-2.920,-0.439)	0.008*	-24.863(-40.361,-9.364)	0.002*	43.138 (7.902,78.375)	0.017*

*Linear regression analysis

**Table 6:** Multivariable-adjusted associations between retinal, GCC/GCIPL, choroidal thickness with age, sex and axial length (AL)

	Retina		GCC		GCIPL		Choroid	
	β(95 % CI)	*p*	β(95 % CI)	*p*	β(95 % CI)	*p*	β(95 % CI)	*p*
Age	-0.139(-0.345,0.067)	0.185	-0.029(-0.121,0.064)	0.540	-0.006(-0.079,0.067)	0.876	-2.290(-4.138,-1.701)	0.000*
AL	-0.626(-3.411,2.160)	0.658	0.748(-0.491,1.987)	0.235	0.050(-0.888,0.989)	0.916	-42.811(-58.655,-26.967)	0.000*
Sex	7.817(1.950,13.684)	0.009*	1.992(-0.640,4.624)	0.137	1.343(-0.843,3.529)	0.227	34.927(0.975,68.880)	0.044*

*Multiple regression analysis

After adjusting for age and AL, gender was still associated with retinal thickness. Retinal thickness was 7.8 um greater in men than in women after adjusting for age and AL. Choroidal thickness was negatively correlated with sex, age, and AL in both linear and multiple regression analyses. Macular choroidal thickness decreased by 2.3 um for each year of life and 42.8 um for each mm of axial length extension. After adjustment for age and AL, men still have a choroid thickness that is 34.9 um greater than in women.

## Discussion

The present study firstly determined the automatic baseline thickness of GCC and GCIPL in the ETDRS grid of healthy Chinese volunteers and assessed the relationship among clinical variables that included sex, age, and AL, and thickness of retina, GCC/GCIPL, and choroid, automatically measured using SS-OCT. The coefficient of variation of GCIPL was found to be the smallest and most stable across different variables. To the best of our knowledge, no previously published paper has elucidated this distribution characteristic in healthy subjects.

The first commercially available SS-OCT (DRI OCT-1, Topcon) uses a longer wavelength-sweeping laser of 1050 nm, which allows for a much higher image acquisition speed and much deeper penetration of the ocular tissue. It enables automatic measurement of retina, local RGC-related layers (GCC and GCIPL), and choroid, and many clinical studies have confirmed its reliability in reproducing ocular tissue measurements, especially for assessment of the choroid [19–24]. SS-OCT was chosen to perform the present study for this reason.

By comparison with total retinal macular thickness, assessment of RGC-related layers provides higher diagnostic power for differentiating between healthy and pathologic eyes. GCC/GCIPL analysis maybe an early and susceptible structural marker for neuronal loss, and estimated macular GCC/GCIPL thickness is considered to be better a diagnostic measure of retinal nerve fiber layer in differentiating preperimetric and perimetric glaucomatous eyes from healthy eyes [8, 25–26]. Previous studies have shown that both GCC and GCIPL thickness can be significantly influenced by ethnicity [27]. The result of this study is of great significance in defining the range of normal variation in healthy Chinese subjects. In these subjects, the mean GCC/GCIPL thickness was 105.3 ± 9.7 um/78.5 ± 6.2 um and varied from 59.6 to 159.4 um/52.4 to 106.0 um. The mean number was significantly lower than one comparative study in healthy Japanese subjects, which used spectral-domain optical coherence tomography (SD-OCT) for measurement [28].

Many studies have demonstrated an independent association between age, sex, AL, and GCC/GCIPL thickness, although the magnitude of this effect is not significant. Older age, female sex, and longer AL are associated with thinner GCC/GCIPL thickness [29–31]. In the present study, a distinct age-related reduction was discovered in GCC thickness, but there was no significant correlation between age and GCC thickness. There seemed to be no obvious change in GCIPL thickness with increasing age and AL. Linear regression analysis showed that AL was negatively correlated with the thickness of GCC, but after adjusting for sex and age, no significant negative correlation was found. The present results also found no significant difference between men and women in the thickness of GCC/GCIPL, which seems somewhat inconsistent with previous studies [29–32]. Table 7 shows the compassion of representative studies concerning GCC and GCIPL thickness in healthy subjects. The biggest difference between our study and previous studies was the area measured. Previously, the most commonly used area for evaluation of GCC/GCIPL thickness was the 14.13 mm^2^ elliptical annulus centered on the fovea. However, in the present study, the modified EDTRS grid (113.04 mm^2^) was used for measurement. In addition, SS-OCT as used here provided significantly better identification of the GCC/GCIPL layer in healthy eyes and reduced extraneous human factors as much as possible. The greater number of women and the differing ethnicity of enrolled volunteers may also partly account for the different results.

**Table 7:** Comparison of representative studies concerning GCC and GCIPL thickness in healthy subjects

Study	Ethnicity	eyes	Sex (female:male)	Age	Axial length	Mean GCC thickness	Mean GCIPL thickness	Measurement equipment	Measurement area
González-López JJ et al. [35]	Spaniard	140	40:30	37 ± 10	-	-	83.8 ± 5.9	Cirrus OCT	6 × 6 × 2 mm elliptical annulus area centered on the fovea
Araie M et al. [28]	Japanese	195	92:103	48.5 ± 16.5	-	123.2 ± 8.5	91.3 ± 6.8	SD-OCT	0.6 mm-diameter circular area corresponding to the 4 central test points of the Humphrey Field Analyzer 24-2 test program
Mwanza JC et al. [29]	Mixed	564	149:133	46.2 ± 16.9	23.94 ± 1.1	-	82.1 ± 6.2	Cirrus HD-OCT	14.13 mm^2^ elliptical annulus area centered on the fovea
Kim NR et al. [32]	Korean	182	109:73	55.5 ± 15.8	24.3 ± 1.4	93.9 ± 7.8	-	RTVue-100 FD-OCT	macular map (MM7), centered 1 mm temporal to the fovea
Tham YC et al. [36]	Chinese	352	164:188	53.6 ± 6.7	24.2 ± 1.2	-	82.8 ± 5.7	Cirrus HD-OCT	14.13 mm^2^ elliptical annulus area centered on the fovea
Current	Chinese	146	99:47	47.9 ± 14.0	23.5 ± 1.1	105.3 ± 9.7	78.5 ± 6.2	SS-OCT	ETDRS grid (6 × 6 mm)

As expected, inter-sex differences and age-related changes were also found in retinal and choroidal thickness. Men had a thicker retina and choroid, and after adjusting for age and AL, men still had 7.8 um greater retinal thickness and 34.9 um greater choroidal thickness than women, which is consistent with the previous results [12]. The main reason for thicker retinal and choroidal thickness in men than in women may be that men have larger eyes. Both retinal and choroidal thickness were found to decrease with increasing age (especially the choroid). Age and AL were negatively related with choroidal thickness, which again confirmed previous findings in the area defined by ETDRS [12, 17, 24]. Song et al. [33] reported that average foveal thickness increased with increasing AL. However, retinal thickness seemed to remain stable with increasing AL in our present study. Given the present study’s larger sample size and wider AL range (20.9–26.9 mm), volunteers were further divided into 5 subgroups, according to AL. There was a very small variation among different AL groups (281.9, 282.3, 282.5, 285.0, 286.2 um in subjects of 20.9–21.99, 22–22.99, 23–23.99, 24–24.99 and >25 mm of AL, respectively). Statistical analysis showed no significant difference among the different subgroups. Ootoet al. [34] have characterized the normal retinal thickness in normal Japanese subjects by use of spectral domain OCT(SD-OCT) and also found no significant correlation of retinal thickness with AL in the ETDRS sectors.

The main strength of the present study (along with the relatively larger sample size and repeatability of measurement procedures) is the application of available built-in software, enabling automated segmentation and thickness measurements of retina, GCC/GCIPL, and choroid. Of course, this study, like others, also had some limitations. One of these was the fact that only healthy Chinese subjects were enrolled. However, our primary purpose was to evaluate the baseline thickness of GCC/GCIPL in the ETDRS grid of such subjects. A further limitation was that the numbers of men and women were not equivalent, as more women were recruited for this study.

## Conclusions

In conclusion, the thickness of GCC/GCIPL in healthy Chinese individuals is similar across different genders, ages, and AL groups in terms of the ETDRS chart. Men’s eyes were found to have thicker retinal and choroid structures. Age and AL also significantly influence the choroidal structure, but not the retina. These data will be of value in diagnosing and monitoring diseases of the ocular fundus and will provide a useful reference for measurements across different races.

## Additional file

Additional file 1:
**STROBE Statement—Checklist of items that should be included in reports of transversal study.** (DOC 88 kb)
