# Supplementary material for: Swept-source optical coherence tomography imaging of macular retinal and choroidal structures in healthy eyes
Source: BMC Ophthalmol. 2015 Sep 17;15:122. doi: 10.1186/s12886-015-0110-3 (PMC4574621; doi:10.1186/s12886-015-0110-3)
Supplement: Additional file 1: — STROBE Statement—Checklist of items that should be included in reports of transversal study. (DOC 88 kb) [file 12886_2015_110_MOESM1_ESM.doc]

STROBE Statement—Checklist of items that should be included in reports of transversal study

|  | Item No | Recommendation |
| --- | --- | --- |
| **Title and abstract** | 1 | (*a*) transversal study |
|  |  | (*b*) We firstly evaluate the differences in the topographic variation between retina, GCC/GCIPL, and choroid with SS-OCT, and the correlation between these parameters with sex, age, and AL in healthy Chinese volunteers. And we found the thickness of GCC/GCIPL in healthy Chinese individuals is similar across different genders, ages, and AL groups in terms of the ETDRS chart. Men’s eyes were found to have thicker retinal and choroid structures. Age and AL also significantly influence the choroidal structure, but not the retina. |
| Introduction | | |
| Background/rationale | 2 | Optical coherence tomography (OCT), as an essential tool in ophthalmology, can noninvasively capture detailed in vivo high resolution images of retinal and choroidal structures, which is useful forvVarious retinal and choroidal pathologies. Qualitative and quantitative analyses of retinal and choroidal structures are critical for the diagnosis and treatment of vitreo-retinal and choroidal diseases. So, we did this study. |
| Objectives | 3 | To measure the baseline GCC/GCIPL thickness with SS-OCT, and to evaluate differences in the topographic variation between retina, GCC/GCIPL, and choroid. |
| Methods | | |
| Study design | 4 | transversal study |
| Setting | 5 | The transversal study included 146 healthy Chinese volunteers, recruited from our hospital staff and the students of Sun Yat-sen University, from January 2014 to June 2014. |
| Participants | 6 | All the study participants were healthy individuals with no history of ocular disease or visual symptoms; aged at least18 years; intraocular pressure (IOP) <21mmHg; normal appearance of optic nerve head; normal anterior chamber angles; and a best-corrected visual acuity (BCVA) of 1.0 or better. Exclusion criteria included IOP>21mmHg; history of intraocular surgery or ocular trauma in the study eye; high myopia or hyperopia (magnitude exceeding±6 diopters of spherical equivalent refraction); |
| Variables | 7 | Only healthy Chinese subjects were enrolled and the numbers of men and women were not equivalent, as more women were recruited for this study. |
| Data sources/ measurement | 8* | *NO* exposed or unexposed groups |
| Bias | 9 | the relatively larger sample size and repeatability of measurement procedures |
| Study size | 10 | Recruited from our hospital staff and the students of Sun Yat-sen University |
| Quantitative variables | 11 | The more the better. |
| Statistical methods | 12 | (*a*) SPSS software version 19.0 (IBM-SPSS, Chicago, Illinois, USA) |
|  |  | (b) Normally distributed data were expressed as mean ± standard deviation (SD). An independent sample t-test was used to compare two different groups where data were normally distributed; a Mann-Whitney U test was used when the data showed a non-parametric distribution. Pearson or Spearman correlation coefficients were used for evaluation of bivariate correlations. For comparison of the thickness of different layers at different subgroups according to age and AL, analysis of variance (ANOVA) or a Kruskal-Wallis test was performed. The relationship between macular retinal and choroidal thickness and sex, age and AL was investigated by linear and stepwise multiple regression analysis. |
|  |  | (*c*) If the missing data was not found, the patient will be excluded. |
|  |  | (*d*) No |
|  |  | (*e*) statistical specialist teachers in our team |
| Results | | |
| Participants | 13* | (a) 146 |
|  |  | (b) No |
|  |  | (c) No |
| Descriptive data | 14* | (a) healthy Chinese volunteers, recruited from our hospital staff and the students of Sun Yat-sen University |
|  |  | (b) No |
| Outcome data | 15* | 146 |
| Main results | 16 | (a) Age, AL, and gender were adjusted. Unadjusted associations and Multivariable-adjusted associations were used. |
|  |  | (*b*) Yes |
|  |  | (*c*) No |
| Other analyses | 17 | No |
| Discussion | | |
| Key results | 18 | 1.Thicknesses of the average macular ganglion cell complex (GCC) and ganglion cell-inner plexiform layer (GCIPL) were 105.3±9.7 and 78.5±6.2um respectively. Neither of them was significantly related with sex, age, or AL. Both showed strong correlations with retinal thickness (r=0.793, p=0.000; r=0.813, p=0.000, respectively) and with similar topographic distributions within the retina.  2.The choroid exhibited completely different patterns of topographic variation. Men had 7.8um thicker retina and 34.9 um thicker choroid than women after adjustment for age and AL (all p<0.05).Age and AL could significantly influence the choroidal thickness but not the retina (all p<0.05). |
| Limitations | 19 | Only healthy Chinese subjects were enrolled and the numbers of men and women were not equivalent, as more women were recruited for this study. |
| Interpretation | 20 | Thickness of GCC/GCIPL in healthy Chinese individuals is similar across different genders, ages, and AL groups in terms of the ETDRS chart. Men’s eyes were found to have thicker retinal and choroid structures. Age and AL also significantly influence the choroidal structure, but not the retina. |
| Generalisability | 21 | These data will be of value in diagnosing and monitoring diseases of the ocular fundus and will provide a useful reference for measurements across different races. |
| Other information | | |
| Funding | 22 | This work was supported in whole or in part by the National Natural Science Foundation of China (81170849, 81371008). |

*Give information separately for exposed and unexposed groups.

**Note:** An Explanation and Elaboration article discusses each checklist item and gives methodological background and published examples of transparent reporting. The STROBE checklist is best used in conjunction with this article (freely available on the Web sites of PLoS Medicine at http://www.plosmedicine.org/, Annals of Internal Medicine at http://www.annals.org/, and Epidemiology at http://www.epidem.com/). Information on the STROBE Initiative is available at www.strobe-statement.org.
